# Adverse COVID-19 experiences and physical and psychological outcomes in patients with lung cancer

**DOI:** 10.1017/S1478951526102004

**Published:** 2026-03-05

**Authors:** Catherine E. Mosher, Stella Snyder, Marcia F. Burns, Gregory A. Durm, DuyKhanh P. Ceppa, Shadia I. Jalal, Thomas J. Birdas, Kenneth A. Kesler, Lawrence H. Einhorn, Nasser Hanna

**Affiliations:** 1 Department of Psychology, Indiana University Indianapolis, Indianapolis, IN, USA; 2 Department of Medicine, Indiana University School of Medicine, Indianapolis, IN, USA; 3 Department of Surgery, Indiana University School of Medicine, Indianapolis, IN, USA

**Keywords:** Lung cancer, COVID-19 pandemic, symptoms, psychological distress, financial hardship

## Abstract

**Objectives:**

Although patients with lung cancer are at high risk of poor outcomes from COVID-19, little is known about the economic and psychosocial impact of the pandemic on this population. This study had 2 objectives: (1) to identify the prevalence of financial and social disruptions and other adverse COVID-19 experiences in socioeconomically diverse patients with lung cancer; and (2) to examine whether these experiences were associated with physical and psychological symptoms.

**Methods:**

Patients with lung cancer (*N* = 191) were recruited from a cancer center in the midwestern U.S. from August 2021 to April 2022 to participate in a cross-sectional survey of COVID-19 experiences and symptoms. Path analyses tested associations between COVID-19 experiences and symptoms, adjusting for sociodemographic and medical covariates.

**Results:**

Prevalent COVID-19 experiences included disrupted interactions with family and friends (66.0%), inability to perform daily routines (38.5%), and financial difficulties (18.5%). Greater financial hardship and disruptions to daily activities and social interactions were associated with higher levels of all physical and psychological symptoms. Endorsing more COVID-19 experiences (e.g., job loss, death of loved ones) was only associated with greater anxiety. Despite prevalent hardships, mean levels of physical and psychological symptoms were within normal ranges, except for elevated fatigue.

**Significance of the results:**

Although adverse COVID-19 experiences were common and related to symptom burden, patients with lung cancer showed notable psychological resilience during the pandemic. Oncology clinicians should consider the impact of COVID-19 experiences when providing financial and support services.

## Introduction

The COVID-19 pandemic had a substantial impact on people’s lives, especially those living with serious illness. Patients with lung cancer, the second most common cancer in the USA (American Cancer Society 2026), have been particularly vulnerable to poor outcomes from COVID-19 (Luo et al. 2020; Centers for Disease Control and Prevention 2025). In a meta-analysis of studies in developed countries, patients with lung cancer and COVID-19 showed a significantly higher mortality rate than patients with other tumors and COVID-19 (42% vs. 24%; Lei et al. 2021). It is also possible that social and economic disruptions due to COVID-19 were more severe for patients with lung cancer than for those with other cancers. Even prior to COVID-19, patients with lung cancer showed higher rates of socioeconomic disadvantage and anxiety and depressive symptoms than patients with other cancers (Linden et al. 2012; Singh and Jemal 2017; Zeilinger et al. 2022).

Limited research has investigated associations between COVID-19-related social and financial disruptions and physical and mental health outcomes in adults with cancer (Perry et al. 2023; Noriega Esquives et al. 2024). These studies enrolled patients with various cancers. For example, survivors of primarily early-stage cancers completed a one-time survey between May 2020 and January 2021 (Noriega Esquives et al. 2024). Results indicated that greater financial hardship, disrupted daily activities and social interactions, and other adverse COVID-19 experiences (e.g., job loss, death of family/friends, COVID-19 hospitalization) were correlated with higher anxiety and depressive symptoms and worse quality of life. Prevalent COVID-19 experiences in this sample were decreased household income (24%) and their spouse or partner losing their job or income (17%) (Otto et al. 2024). A similar one-time survey of cancer survivors was conducted from June 2021 to March 2022 (Perry et al. 2023). Participants who reported a greater number of adverse COVID-19 experiences (e.g., job loss, death of family/friends, COVID-19 hospitalization) experienced higher levels of COVID-related anxiety and depressive symptoms and worse quality of life.

Findings on mental health outcomes in cancer populations during the pandemic are mixed (Ballesteros et al. 2023; Almeida et al. 2024). In a review of studies published through January 2021, patients with cancer typically had high levels of anxiety and depressive symptoms (Almeida et al. 2024). However, longitudinal studies did not show consistent differences in distress between pre- and post-pandemic periods or increased distress compared to the general population during the pandemic. It should be noted that studies varied in their self-report methods, country of origin, and timepoints, as COVID-19 cases and government-imposed restrictions fluctuated. It is also possible that certain patient subgroups, such as those with lung cancer, were at higher risk of distress. Indeed, one large German study in 2021 found that adults with lung cancer had higher rates of clinically meaningful anxiety and depressive symptoms than other cancer groups (Doege et al. 2025). However, results of a U.S. study suggested that the pandemic did not impact distress in patients with advanced lung cancer (Petrillo et al. 2022). Specifically, baseline depressive symptoms and quality of life did not differ between patients with advanced lung cancer enrolled in an early palliative care trial pre-pandemic (March 2018 to January 2019) vs. during the pandemic (January 2020 to January 2021). Unmarried patients, however, showed lower quality of life during the pandemic, pointing to possible effects of decreased social engagement and loneliness.

Little research has examined both physical and psychological symptoms in relation to COVID-19 experiences in cancer populations. Additionally, limited research has focused on the experiences of adults with lung cancer (Petrillo et al. 2022; Gomes et al. 2023), a population at high risk of adverse outcomes from COVID-19 (Luo et al. 2020; Centers for Disease Control and Prevention 2025). To address gaps in the literature, a cross-sectional survey of COVID-19 experiences – including financial and social disruptions – and physical and psychological symptoms was conducted in a socioeconomically diverse sample of U.S. patients with lung cancer from August 2021 through May 2022. The present study had 2 aims: (1) to identify the prevalence of certain financial and social disruptions and other adverse COVID-19 experiences (e.g., COVID-19 hospitalization, death of family members) in patients with lung cancer; and (2) to examine whether financial hardship, disruptions in activities and social interactions, and other adverse COVID-19 experiences were linked to physical symptoms (pain, fatigue, sleep disturbance) and psychological symptoms (anxiety, depressive symptoms, perceived social isolation). We hypothesized that a greater number of adverse COVID-19 experiences would be associated with higher symptom levels.

## Method

### Participants and procedure

Following Indiana University institutional review board approval, study recruitment took place from August 2021 through May 2022. Study inclusion criteria were as follows: (1) received care at the Indiana University Simon Comprehensive Cancer Center for lung cancer of any stage; (2) at least 18 years of age; (3) English fluency; and (4) no significant psychiatric or cognitive impairment based on investigator judgment and a 6-item cognitive screening (Callahan et al. 2002).

Potential participants were identified through medical chart review and oncologist consultation. Patients were then mailed a study brochure and consent form and called for eligibility screening and verbal informed consent. Consenting patients completed a one-time, 30-minute survey either via postal mail or online based on their preference. To increase the response rate, reminder emails and phone calls were made, and patients were mailed a $25 gift card for survey return. Information on participants’ COVID-19-related beliefs and behaviors (e.g., vaccination, mask wearing) were published in a separate report (Burns et al. 2025).

### Measures

#### Financial hardship

The Financial Hardship measure assessed financial difficulties since the onset of the COVID-19 pandemic (Saez-Clarke et al. 2023). The five items were rated on 5-point scales from 0 (*strongly disagree*) to 4 (*strongly agree*). A sample item is: “I have not been able to adequately provide for others I financially support.” Items were averaged with higher scores indicating greater financial hardship. The measure has evidence of reliability and validity in patients with cancer (Perry et al. 2023; Saez-Clarke et al. 2023). Internal consistency reliability in the present research was 0.86.

#### Disruptions to daily activities and social interactions

The Disruptions to Daily Activities and Social Interactions measure assessed difficulties in functional and social domains since the onset of the COVID-19 pandemic (Saez-Clarke et al. 2023). The five items were rated on 5-point scales from 0 (*strongly disagree*) to 4 (*strongly agree*). A sample item is: “I have been unable to perform my typical daily routines (e.g., work, physical activity, leisure activity).” Items were averaged with higher scores indicating greater disruptions. The measure has evidence of reliability and validity in patients with cancer (Perry et al. 2023; Saez-Clarke et al. 2023). Internal consistency reliability in the present research was 0.79.

#### Adverse COVID-19 experiences

Patients responded to 9 yes/no questions assessing adverse COVID-19 experiences that have been tested in patients with cancer (Perry et al. 2023). Sample items include: “If you tested positive for COVID-19, were you hospitalized?,” “Did a family member or a member of your household die of COVID-19?,” and “Did you lose your job or primary source of income due to COVID-19?” Items were summed to indicate the total number of adverse COVID-19 experiences.

#### Physical and psychological symptoms

Four-item Patient-Reported Outcomes Measurement Information System (PROMIS) measures were used to assess the following symptoms: fatigue, sleep disturbance, anxiety, depressive symptoms, and perceived social isolation (Cella et al. 2007, 2010; Choi et al. 2010; Yu et al. 2012; Pilkonis et al. 2014). In addition, a 3-item PROMIS measure of pain intensity was used (Cella et al. 2010). For each measure, *T*‐scores were calculated relative to the U.S. general population, with a mean of 50 and a standard deviation of 10. Based on published threshold scores, *T*-scores < 50 for fatigue measures and *T*-scores < 55 for sleep disturbance, anxiety, depression, and social isolation measures are considered within the normal range (Cella et al. 2014; Northwestern University 2025). PROMIS measures have evidence of reliability and validity in patients with cancer (Yost et al. 2011; Wagner et al. 2015; Jensen et al. 2017; Lee et al. 2023). Internal consistency reliabilities in the present research ranged from 0.87 to 0.95.

#### Demographic and medical factors

Patients reported standard demographics and their medical conditions on an 8-item checklist (e.g., asthma, hypertension, diabetes, heart failure) (Kroenke et al. 2009, 2010). Patients also rated their functional status over the past month on a 5-point Eastern Cooperative Oncology Group (ECOG) scale ranging from 0 (“*Normal with no limitations”*) to 4 (“*Pretty much bedridden, rarely out of bed”*) (Bauer et al. 2002). Additionally, smoking was assessed with 2 items from the Centers for Disease Control and Prevention’s Behavioral Risk Factor Surveillance System (BRFSS; Centers for Disease Control and Prevention 2007). Age, gender, and cancer-related information (e.g., disease stage, treatments) were extracted from medical records.

### Data analysis

For Aim 1, the prevalence of each financial or social disruption was computed. For Aim 2, path analyses were conducted in Mplus Version 8.11 (Muthén and Muthén 2017) to test the hypothesis that greater financial hardship, disruptions in activities and social interactions, and other adverse COVID-19 experiences would be associated with higher symptom levels. All path analyses were estimated using maximum likelihood methods and were restricted to observed variables. Six models tested whether the three predictors were associated with physical symptoms (pain, fatigue, sleep disturbance) and psychological symptoms (anxiety, depressive symptoms, social isolation). Predictor variables were standardized as *z*-scores, and outcome variables were converted to *T*-scores (*M* = 50, *SD* = 10) to facilitate comparisons with general population norms.

Covariates were established correlates of symptoms in patients with cancer (Mao et al. 2007; Linden et al. 2012; Reilly et al. 2013; Thomas et al. 2014; Braamse et al. 2016; Erim et al. 2019; Cohee et al. 2020). Models adjusted for the following covariates: age, gender, education, income, number of medical comorbidities, cancer treatment status (0 = not receiving, 1 = received treatment in past 4 weeks), and cancer stage (1 = early, 2 = advanced). Early-stage disease was defined as stage I–II non-small cell lung cancer or limited-stage small cell lung cancer; advanced stage included Stage III–IV non-small cell or extensive-stage small cell lung cancer. Residual covariances among outcomes were freely estimated. All models were just-identified, rendering global fit indices (e.g., Root Mean Square Error of Approximation [RMSEA], Comparative Fit Index [CFI]) uninterpretable.

## Results

Of the 354 patients who were sent recruitment materials, 78 refused study participation, 49 could not be reached via phone, and 4 were deceased. The majority (74%) of reached patients completed the eligibility screening, and all eligible patients consented. Of the 212 consenting patients, 17 were lost to follow-up, 2 withdrew, 1 died, and 192 patients (91%) returned the survey. One survey was excluded from analyses due to substantial missingness (80% of the survey was incomplete); thus, 191 surveys were analyzed.

Supplemental Table 1 shows the sample’s demographic and medical characteristics. The majority were non-Hispanic white (89.0%) and female (62.3%), and a wide range of education and income levels were represented. On average, patients had been living with their lung cancer diagnosis for 2 years, and 42.4% were currently receiving cancer treatment. Supplemental Table 2 shows descriptive statistics for main study variables. All symptoms were within normal ranges, except for fatigue, which exceeded the cutpoint of 50 for oncology patients (Cella et al. 2014). Supplemental Table 3 shows the prevalence of 9 adverse COVID-19 experiences. While only 13.6% of the sample had tested positive for COVID-19, 8.9% had experienced the death of a family member from COVID-19, and 16.2% reported a decline in household income since the onset of the pandemic.

**Figure 1.**
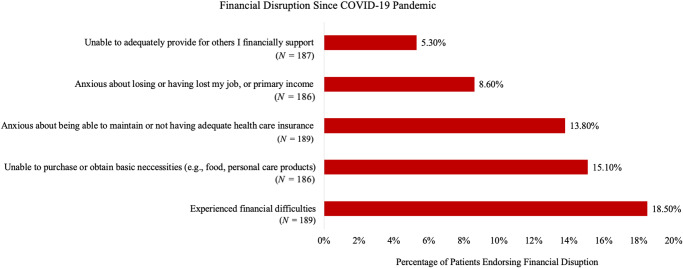
Financial disrupution since COVID-19 pandemic.

**Figure 2.**
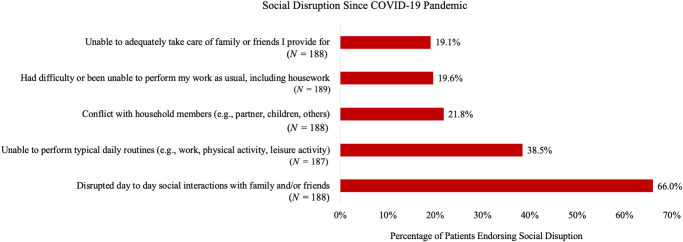
Social disruption since COVID-19 pandemic.

For Aim 1, Figs. 1 and 2 show the prevalence of specific financial and social disruptions attributable to COVID-19, respectively. The most common financial disruptions were the experience of “financial difficulties” (18.5%) and being “unable to purchase or obtain basic necessities,” such as food (15.1%). The most common social disruptions were disrupted “day to day social interactions with family and/or friends” (66.0%) and being “unable to perform typical daily routines (e.g., work, physical activity, leisure activity)” (38.5%).

For Aim 2, path analyses were conducted to test the hypothesis that more adverse COVID-19 experiences would be linked to higher symptom levels, controlling for demographic and medical covariates. First, greater financial hardship was significantly associated with greater pain (β = 0.21, *p* = .004), fatigue (β = 0.21, *p* = .006), and sleep disturbance (β = 0.36, *p* < .001), as well as elevated anxiety (β = 0.47, *p* < .001), depressive symptoms (β = 0.38, *p* < .001), and social isolation (β = 0.36, *p* < .001; see Table 1). Greater medical comorbidities and lower income were linked to worse physical symptoms, and greater comorbidities were associated with higher social isolation. Residual correlations among physical and psychological symptoms were significant, supporting their inclusion in joint models. *R*^2^ values for physical symptom models ranged from 0.23 to 0.27, and those for psychological symptom models ranged from 0.24 to 0.27.

**Table 1.** Associations of financial hardship with physical and psychological symptoms

Outcome variable	*R*^2^		β	SE	*z*	*p*
Physical symptoms (*N* = 177)						
Pain	0.27**	Financial hardship	0.21	0.07	2.86	<.01**
		Age	−0.10	0.07	−1.36	.17
		Gender	0.05	0.07	0.70	.48
		Comorbidities	0.25	0.07	3.65	<.01**
		Income	−0.17	0.08	−2.13	.03*
		Education	−0.12	0.08	−1.45	.15
		Cancer treatment status	−0.00	0.08	−0.04	.97
		Cancer stage	−0.02	0.08	−0.29	.78
Fatigue	0.23**	Financial hardship	0.21	0.07	2.76	<.01**
		Age	−0.06	0.07	−0.88	.38
		Gender	0.01	0.07	0.09	.93
		Comorbidities	0.27	0.07	3.85	<.01**
		Income	−0.18	0.08	−2.17	.03*
		Education	−0.03	0.08	−0.39	.70
		Cancer treatment status	0.06	0.08	0.76	.45
		Cancer stage	0.05	0.08	0.62	.54
Sleep disturbance	0.26**	Financial hardship	0.36	0.07	5.22	<.01**
		Age	−0.08	0.07	−1.13	.26
		Gender	−0.03	0.07	−0.38	.71
		Comorbidities	0.14	0.07	2.03	.04
		Income	−0.24	0.08	−2.97	<.01**
		Education	0.07	0.08	0.81	.42
		Cancer treatment status	−0.02	0.08	−0.23	.82
		Cancer stage	0.01	0.08	0.15	.89
Psychological symptoms (*N* = 177)						
Anxiety	0.24**	Financial hardship	0.47	0.07	6.84	<.01**
		Age	0.03	0.07	0.45	.65
		Gender	0.13	0.07	1.90	.06
		Comorbidities	0.07	0.07	0.91	.36
		Income	0.00	0.08	0.02	.98
		Education	0.03	0.08	0.41	.68
		Cancer treatment status	0.03	0.08	0.41	.68
		Cancer stage	−0.01	0.08	−0.07	.94
Depressive symptoms	0.26**	Financial hardship	0.38	0.07	5.48	<.01**
		Age	0.05	0.07	0.76	.45
		Gender	0.05	0.07	0.74	.46
		Comorbidities	0.13	0.07	1.84	.07
		Income	−0.17	0.08	−2.12	.03*
		Education	0.00	0.08	0.02	.98
		Cancer treatment status	0.06	0.08	0.74	.46
		Cancer stage	−0.01	0.08	−0.15	.88
Social isolation	0.27**	Financial hardship	0.36	0.07	5.16	<.01**
		Age	−0.03	0.07	−0.41	.68
		Gender	0.11	0.07	1.64	.10
		Comorbidities	0.26	0.07	3.85	<.01**
		Income	−0.13	0.08	−1.59	.11
		Education	0.09	0.08	1.10	.27
		Cancer treatment status	0.08	0.08	0.97	.33
		Cancer stage	0.00	0.08	0.01	.99

*Note*:

* *p <* .05. **p < .01. Gender coded as 0 = male, 1 = female. Cancer treatment status coded as 0 = not in active treatment, 1 = received treatment ≤ 4 weeks ago. Cancer stage coded as 1 = early-stage disease, 2 = advanced stage disease. All outcomes used standardized *T*-scores (*M* = 50, *SD* = 10). Standardized regression coefficients are reported.

Second, greater disruptions to daily activities and social interactions were significantly correlated with more severe pain (β = 0.17, *p* = .01), fatigue (β = 0.29, *p* < .001), sleep disturbance (β = 0.28, *p* < .001), anxiety (β = 0.52, *p* < .001), depressive symptoms (β = 0.41, *p* < .001), and social isolation (β = 0.48, *p* < .001; see Table 2). Among the covariates, lower income was associated with higher levels of all physical symptoms, and greater medical comorbidities were associated with higher pain and fatigue. Lower levels of education were also related to greater pain, and younger age was linked to greater sleep disturbance. For social isolation, significant correlates included lower income, younger age, and greater comorbidities. Lower income was also related to greater depressive symptoms. Residual covariances among outcomes were significant in all models. *R*^2^ values for physical symptom models ranged from 0.24 to 0.27, and those for psychological symptom models ranged from 0.30 to 0.38.

**Table 2.** Associations of disruptions to daily activities and social interactions with physical and psychological symptoms

Outcome variable	*R*^2^		β	SE	*z*	*p*
Physical symptoms (*N* = 179)						
Pain	0.26**	Social disruptions	0.17	0.07	2.59	.01*
		Age	−0.13	0.07	−1.82	.07
		Gender	0.03	0.07	0.44	.66
		Comorbidities	0.25	0.07	3.53	< .01**
		Income	−0.19	0.08	−2.45	.01*
		Education	−0.17	0.08	−2.10	.04*
		Cancer treatment status	−0.02	0.08	−0.31	.76
		Cancer stage	0.00	0.08	0.03	.97
Fatigue	0.27**	Social disruptions	0.29	0.07	4.43	< .01**
		Age	−0.12	0.07	−1.74	.08
		Gender	−0.04	0.07	−0.58	.57
		Comorbidities	0.23	0.07	3.21	< .01**
		Income	−0.24	0.08	−3.03	< .01**
		Education	−0.06	0.08	−0.78	.44
		Cancer treatment status	0.02	0.08	0.20	.84
		Cancer stage	0.08	0.08	0.99	.32
Sleep disturbance	0.24**	Social disruptions	0.28	0.07	4.23	< .01**
		Age	−0.16	0.07	−2.23	.03*
		Gender	−0.05	0.07	−0.71	.48
		Comorbidities	0.11	0.07	1.56	.12
		Income	−0.32	0.08	−4.05	< .01**
		Education	−0.02	0.08	−0.18	.86
		Cancer treatment status	−0.06	0.08	−0.74	.46
		Cancer stage	0.05	0.08	0.66	.51
Psychological symptoms (*N* = 179)						
Anxiety	0.32**	Social disruptions	0.52	0.06	9.10	< .01**
		Age	−0.09	0.07	−1.38	.17
		Gender	0.05	0.06	0.83	.40
		Comorbidities	−0.04	0.07	−0.55	.59
		Income	−0.13	0.08	−1.75	.08
		Education	−0.05	0.08	−0.66	.51
		Cancer treatment status	−0.01	0.08	−0.18	.85
		Cancer stage	0.03	0.08	0.45	.66
Depressive symptoms	0.30**	Social disruptions	0.41	0.06	6.67	< .01**
		Age	−0.05	0.07	−0.78	.44
		Gender	−0.01	0.07	−0.19	.85
		Comorbidities	0.05	0.07	0.66	.51
		Income	−0.29	0.08	−3.79	< .01**
		Education	−0.06	0.08	−0.70	.48
		Cancer treatment status	0.01	0.08	0.18	.86
		Cancer stage	0.01	0.08	0.14	.89
Social isolation	0.38**	Social disruptions	0.48	0.06	8.59	< .01**
		Age	−0.14	0.06	−2.22	.03*
		Gender	0.03	0.06	0.55	.58
		Comorbidities	0.15	0.07	2.23	.03*
		Income	−0.27	0.07	−3.72	< .01**
		Education	0.05	0.07	0.62	.54
		Cancer treatment status	0.03	0.07	0.35	.73
		Cancer stage	0.02	0.07	0.21	.83

*Note*:

* *p < .*05. **p < .01. Gender coded as 0 = male, 1 = female. Cancer treatment status coded as 0 = not in active treatment, 1 = received treatment ≤ 4 weeks ago. Cancer stage coded as 1 = early-stage disease, 2 = advanced stage disease. All outcomes used standardized *T*-scores (*M* = 50, *SD* = 10). Standardized regression coefficients are reported.

Finally, a higher number of adverse COVID-19 experiences was significantly correlated with greater anxiety (β = 0.21, *p* = .003), but was not significantly related to depressive symptoms, social isolation, or physical symptoms (all *p*s > .05; see Table 3). Across models, lower income and greater comorbidities were associated with worse physical and psychological symptoms, except for anxiety. Residual covariances among outcomes remained significant across all models. *R*^2^ values for physical symptom models ranged from 0.17 to 0.24, and those for psychological symptom models ranged from 0.11 to 0.19.

**Table 3.** Associations of adverse COVID-19 experiences with physical and psychological symptoms

Outcome variable	*R*^2^		β	SE	*z*	*p*
Physical Symptoms (*N* = 183)						
Pain	0.24**	COVID − 19 experiences	0.06	0.07	0.87	.38
		Age	−0.13	0.07	−1.84	.07
		Gender	0.06	0.07	0.83	.41
		Comorbidities	0.28	0.07	4.12	< .01**
		Income	−0.22	0.08	−2.73	.01*
		Education	−0.15	0.08	−1.88	.06
		Cancer treatment status	0.00	0.08	0.01	.99
		Cancer stage	− 0.02	0.08	−0.24	.81
Fatigue	0.19**	COVID − 19 experiences	0.08	0.07	1.12	.26
		Age	−0.10	0.07	−1.31	.19
		Gender	−0.01	0.07	−0.13	.90
		Comorbidities	0.29	0.07	4.12	< .01**
		Income	−0.24	0.08	−2.88	< .01**
		Education	−0.05	0.08	−0.62	.54
		Cancer treatment status	0.08	0.08	1.03	.30
		Cancer stage	0.04	0.08	0.51	.61
Sleep disturbance	0.17**	COVID − 19 experiences	0.10	0.07	1.36	.17
		Age	−0.14	0.07	−1.96	.05
		Gender	−0.02	0.07	−0.29	.77
		Comorbidities	0.18	0.07	2.46	.01*
		Income	−0.33	0.08	−4.08	< .01**
		Education	−0.00	0.08	−0.02	.98
		Cancer treatment status	0.01	0.08	0.08	.94
		Cancer stage	0.02	0.08	0.20	.84
Psychological symptoms (*N* = 183)						
Anxiety	0.11*	COVID − 19 experiences	0.21	0.07	2.96	< .01**
		Age	−0.04	0.08	−0.56	.58
		Gender	0.11	0.07	1.50	.13
		Comorbidities	0.08	0.08	1.01	.31
		Income	−0.14	0.09	−1.67	.10
		Education	−0.06	0.09	−0.69	.49
		Cancer treatment status	0.09	0.09	1.06	.29
		Cancer stage	0.00	0.09	0.05	.96
Depressive symptoms	0.15**	COVID − 19 experiences	0.05	0.07	0.75	.45
		Age	−0.03	0.08	−0.37	.71
		Gender	0.03	0.07	0.46	.65
		Comorbidities	0.16	0.07	2.10	.04*
		Income	−0.27	0.08	−3.26	< .01**
		Education	−0.08	0.09	−0.88	.38
		Cancer treatment status	0.09	0.08	1.02	.31
		Cancer stage	− 0.00	0.08	−0.05	.96
Social isolation	0.19**	COVID − 19 experiences	0.13	0.07	1.87	.06
		Age	−0.11	0.07	−1.47	.14
		Gender	0.09	0.07	1.31	.19
		Comorbidities	0.27	0.07	3.87	< .01**
		Income	−0.24	0.08	−2.93	< .01**
		Education	0.00	0.08	0.02	.99
		Cancer treatment status	0.10	0.08	1.22	.22
		Cancer stage	0.03	0.08	0.32	.75

*Note*:

* *p < .*05. **p < .01. COVID-19 experiences ranged from 0 to 7 adverse experiences. Gender coded as 0 = male, 1 = female. Cancer treatment status coded as 0 = not in active treatment, 1 = received treatment ≤ 4 weeks ago. Cancer stage coded as 1 = early-stage disease, 2 = advanced stage disease. All outcomes used standardized *T*-scores (*M* = 50, *SD* = 10). Standardized regression coefficients are reported.

## Discussion

Although patients with lung cancer remain at high risk for adverse COVID-19 outcomes (Centers for Disease Control and Prevention 2025), limited research has documented the psychosocial and economic impact of the pandemic on this population (Petrillo et al. 2022; Gomes et al. 2023). In this socioeconomically diverse sample of adults with lung cancer, many endorsed disrupted interactions with family and friends (66.0%) and being unable to perform daily routines (38.5%) during the pandemic. A significant proportion also reported financial difficulties (18.5%) and increased challenges in affording basic necessities (15.1%) since the onset of the pandemic. Despite these hardships, mean levels of physical and psychological symptoms fell within normative ranges, except for elevated fatigue. However, greater financial hardship and disrupted daily activities and social interactions were associated with higher levels of all physical and psychological symptoms. Other COVID-19 experiences (e.g., decreased income, death of loved ones) only showed a significant association with greater anxiety. Overall, while COVID-19 experiences were related to symptom burden, patients with lung cancer demonstrated notable psychological resilience when faced with multiple adversities.

Most COVID-19 experiences in this sample (e.g., decreased income) did not deviate markedly from those reported by patients with various cancers during a similar period (2021–2022) (Perry et al. 2023). However, compared to the previous study, this sample had higher rates of family members testing positive for COVID-19 (43.5% vs. 21.9%) and dying of COVID-19 (8.9% vs. 3.6%). These findings may be related to lower levels of education and income in the current sample, which are associated with COVID-19 protective behaviors and healthcare access (McMaughan et al. 2020; Papageorge et al. 2021; Folayan et al. 2023). Additionally, smoking is a risk factor for lung cancer and COVID-19 mortality (Patanavanich et al. 2022; Leiter et al. 2023) that aggregates within families (Wang et al. 2022).

In this study, both financial hardship and disrupted daily activities and social interactions due to COVID-19 were correlated with higher levels of common physical and psychological symptoms. While prior research has linked similar COVID-19 experiences to increased distress and reduced global quality of life in patients with diverse cancers (Perry et al. 2023; Noriega Esquives et al. 2024), this study expands this analysis to include common physical symptoms and perceived social isolation. Current findings suggest that COVID-19-related stressors have a broad impact on physical and psychosocial well-being in patients with lung cancer.

Endorsing a greater number of adverse COVID-19 experiences was associated with higher anxiety levels, but not with other psychological or physical symptoms. On average, participants reported 2 adverse COVID-19 experiences, with the most common being knowing a family member or another person (friend, neighbor, or coworker) who had tested positive. Experiences more closely linked to depressive symptoms or social isolation (e.g., death of a loved one) or to physical symptoms (e.g., hospitalization due to COVID-19) were reported by fewer participants, which may have limited the ability to detect associations with these outcomes.

Physical and psychological symptom levels for this sample were within the normal range, except for fatigue. On average, participants were older adults (*M* = 66 years) who had been living with lung cancer for 2 years. Having potentially adapted to preexisting limitations in daily activities, older patients may demonstrate greater capacity to cope with the additional restrictions introduced by the COVID-19 pandemic. Conversely, elevated fatigue in this sample likely reflects both its high prevalence in cancer populations (Bower 2014) and the fact that 42% of participants were undergoing active cancer treatment. Additionally, patients with lower income and greater medical comorbidities showed higher symptom levels, as found in prior research (Mao et al. 2007; Thomas et al. 2014; Cohee et al. 2020).

Limitations of this study include the cross-sectional design, which precludes the evaluation of temporal relationships among variables. Additionally, although a range of COVID-19 experiences were assessed, disruptions in healthcare resulting from the pandemic were not captured. Finally, participants were primarily non-Hispanic white and recruited from a cancer center in the midwestern USA. However, the study response rate was high, and a wide range of income and education levels were represented.

In conclusion, many patients with lung cancer reported adverse COVID-19 experiences, including financial hardship and social difficulties, which were associated with greater symptom burden. These findings underscore the importance of accounting for the impacts of COVID-19 when delivering financial assistance and supportive services to this population. Notably, despite experiencing substantial hardship, the present sample demonstrated considerable psychological resilience, warranting further investigation. Given that patients with lung cancer remain at elevated risk for adverse COVID-19 outcomes (Centers for Disease Control and Prevention 2025), future research should examine the long-term health, psychosocial, and economic consequences of the pandemic for this population. Such research may inform health care policy and the development of targeted clinical interventions.

## Supporting information

10.1017/S1478951526102004.sm001 Mosher et al. supplementary material Mosher et al. supplementary material

## Supplementary material

The supplementary material for this article can be found at https://doi.org/10.1017/S1478951526102004
