# Supplementary material for: Adverse COVID-19 experiences and physical and psychological outcomes in patients with lung cancer
Source: Palliat Support Care. 2026 Mar 5;24:e80. doi: 10.1017/S1478951526102004 (PMC13166432; doi:10.1017/S1478951526102004)
Supplement: Mosher et al. supplementary material [file S1478951526102004sup001.docx]

| **Supplemental Table 1.** |  |
| --- | --- |
| ***Demographic and Medical Characteristics (N = 191)*** |  |
| Age (years) |  |
| Mean (SD) | 66.4 (9.9) |
| Range | 34-91 |
| Gender, *n* (%) |  |
| Female | 119 (62.3%) |
| Male | 72 (37.7%) |
| Race and ethnicity^a^, *n* (%) |  |
| African American or Black | 11 (5.8%) |
| Asian | 2 (1.0%) |
| Hispanic or Latinx | 4 (2.1%) |
| non-Hispanic White | 170 (89.0%) |
| Native American or Alaska Native | 3 (1.6%) |
| Native Hawaiian or Other Pacific Islander | 1 (0.5%) |
| Not Disclosed | 2 (1.0%) |
| Employment status, *n* (%) |  |
| Employed full or part-time | 51 (26.7%) |
| Homemaker | 6 (3.1%) |
| Retired | 106 (55.5%) |
| Unemployed | 28 (14.7%) |
| Household income, *n* (%) |  |
| Less than $21,000 | 24 (12.6%) |
| $21,000 - $30,999 | 21 (11.0%) |
| $31,000 - $50,999 | 46 (24.1%) |
| $51,000 - $99,999 | 46 (24.1%) |
| $100,000 or more | 46 (24.1%) |
| Not disclosed | 8 (4.2%) |
| Number of household members, including self |  |
| Mean (SD) | 2.17 (1.0) |
| Range | 1-7 |
| Education level, *n* (%) |  |
| Less than grade 12 | 15 (7.8%) |
| Grade 12 or GED (High school graduate) | 47 (24.6%) |
| College 1 to 3 years | 62 (32.5%) |
| College 4 years or more (College graduate) | 37 (19.4%) |
| Graduate school | 29 (15.2%) |
| Not disclosed | 1 (0.5%) |
| Relationship status, *n* (%) |  |
| Married/living with a partner | 122 (63.9%) |
| Unpartnered | 69 (36.1%) |
| Cancer stage, *n* (%) |  |
| Early (stage I-II NSCLC, limited SCLC) | 90 (47.1%) |
| Advanced (stage III-IV NSCLC, extensive SCLC) | 101 (52.9%) |
| Time since diagnosis (years) |  |
| Mean (SD) | 1.98 (2.87) |
| Range | 0-22 |
| Currently undergoing cancer treatment^b^, *n* (%) |  |
| Yes | 81 (42.4%) |
| Current cancer treatment, *n* (%) |  |
| Chemotherapy | 26 (13.6%) |
| Radiation | 9 (4.7%) |
| Chemoradiation | 3 (1.6%) |
| Targeted therapy | 40 (20.9%) |
| Immunotherapy | 31 (16.2%) |
| Current smoking, *n* (%) |  |
| Yes | 30 (15.7%) |
| Patient-reported ECOG score^c^ |  |
| Mean (SD) | 1.02 (0.91) |
| Range | 0-4 |
| Number of comorbidities |  |
| Mean (SD) | 1.52 (1.24) |
| Range | 0-7 |
| *Note.* ECOG = Eastern Cooperative Oncology Group; NSCLC = non-small cell lung cancer; SCLC = small cell lung cancer. | |
| ^a^ Patients could report multiple races or ethnicities. |  |
| ^b^ Treatment received < 4 weeks ago. |  |
| ^c^ Higher scores indicate greater functional impairment. |  |

| **Supplemental Table 2.** |  |
| --- | --- |
| ***Descriptive Statistics for Main Study Variables*** |  |
| PROMIS Pain Intensity |  |
| Mean *T*-score^a^ (SD) | 51.37 (11.85) |
| Range | 36.30-80.70 |
| PROMIS Fatigue |  |
| Mean *T*-score^a^ (SD) | 53.32 (9.22) |
| Range | 33.70-75.80 |
| PROMIS Sleep Disturbance |  |
| Mean *T*-score^a^ (SD) | 50.71 (8.41) |
| Range | 32.00-73.30 |
| PROMIS Anxiety |  |
| Mean *T*-score^a^ (SD) | 50.86 (8.59) |
| Range | 40.50-75.30 |
| PROMIS Depression |  |
| Mean *T*-score^a^ (SD) | 49.93 (8.72) |
| Range | 41.00-73.10 |
| PROMIS Social Isolation |  |
| Mean *T*-score^a^ (SD) | 44.45 (9.18) |
| Range | 34.80-70.50 |
| Financial Hardship |  |
| Mean (SD) | 1.01 (0.89) |
| Range | 0.00-3.60 |
| Disruptions to Daily Activities and Social Interactions |  |
| Mean (SD) | 1.62 (0.93) |
| Range | 0.00-4.00 |
| Number of adverse COVID-19 experiences |  |
| Mean (SD) | 1.82 (1.31) |
| Range | 0.00-7.00 |
| *Note.* PROMIS = Patient-Reported Outcomes Measurement Information System. *N*s = 183-191. | |
| ^a^ The general U.S. population mean *T*-score is 50 (SD=10). |  |

| **Supplemental Table 3.**  ***Prevalence of Adverse COVID-19 Experiences (N = 191)*** *n* (%) | |
| --- | --- |
| Positive for COVID-19, if tested | 26 (13.6) |
| Hospitalized for COVID-19 | 7 (3.7) |
| Family member or member of household tested positive for COVID-19 | 83 (43.5) |
| Family member or member of household died of COVID-19 | 17 (8.9) |
| Friend, co-worker, or neighbor tested positive for COVID-19 | 128 (67.0) |
| Friend, co-worker, or neighbor died of COVID-19 | 42 (22.0) |
| Lost job or primary source of income due to COVID-19 | 4 (2.1) |
| Spouse or partner lost job or primary source of income due to COVID-19 | 9 (4.7) |
| Household income decreased due to COVID-19 | 31 (16.2) |
